# The Leptospiral General Secretory Protein D (GspD), a secretin, elicits complement-independent bactericidal antibody against diverse *Leptospira* species and serovars

**DOI:** 10.1016/j.jvacx.2021.100089

**Published:** 2021-02-23

**Authors:** EJA. Schuler, RT. Marconi

**Affiliations:** Department of Microbiology and Immunology, Virginia Commonwealth University Medical Center, 1112 E Clay St., Richmond, VA 23298, USA

**Keywords:** Canine, GspD, Leptospirosis, Secretin, Spirochetes, Type 2 secretion

## Abstract

Leptospirosis, the most common zoonotic infection worldwide, is a multi-system disorder affecting the kidney, liver, and lungs. Infections can be asymptomatic, self-limiting or progress to multi-organ system failure and pulmonary hemorrhage. The incidence of canine and human leptospirosis is steadily increasing worldwide. At least sixty-four *Leptospira* species and several hundred lipopolysaccharide-based serovars have been defined. Preventive vaccines are available for use in veterinary medicine and limited use in humans in some countries. All commercially available vaccines are bacterin formulations that consist of a combination of laboratory cultivated strains of different lipopolysaccharide serotypes. The development of a broadly protective subunit vaccine would represent a significant step forward in efforts to combat leptospirosis in humans, livestock, and companion animals worldwide. Here we investigate the potential of General secretory protein D (GspD; LIC11570), a secretin, to serve as a possible antigen in a multi-valent vaccine formulation. GspD is conserved, expressed *in vitro,* antigenic during infection and elicits antibody with complement independent bactericidal activity. Importantly, antibody to GspD is bactericidal against diverse *Leptospira* species of the P1 subclade. Epitope mapping localized the bactericidal epitopes to the N-terminal N0 domain of GspD. The data within support further exploration of GspD as a candidate for inclusion in a next generation multi-protein subunit vaccine.

## 1 Introduction

Leptospirosis is a potentially life-threatening multi-systemic zoonotic infection of veterinary and human concern that occurs worldwide [1], [2]. The incidence of leptospirosis is highest in tropical and subtropical regions where socioeconomic conditions are poor. The genus *Leptospira* consists of at least 64 pathogenic and saprophytic species that form two pathogenic and saprophytic clades (P and S, respectively). These clades can be further divided into subclades (P1, P2, S1 and S2) [3]. While the virulence potential of many *Leptospira* species remains to be determined, most well characterized pathogenic species belong to the P1 subclade [3]. *Leptospira* have also been classified based on the antigenic properties of their lipopolysaccharide (LPS) resulting in the designation of hundreds of serovars.

*Leptospira* are maintained in nature by a wide array of reservoir hosts [3]. As a consequence of the ability of pathogenic species to establish chronic colonization of the kidneys, leptospires are continually shed in urine. Contact with animals (livestock, wildlife and companion), contaminated soil, and water serve as common sources of infection [4]. The severity of leptospirosis can range from asymptomatic colonization to mild or serious infection with life-threatening clinical manifestations including compromised kidney and liver function and pulmonary hemorrhage [2], [5]. Over 1,000,000 cases of human leptospirosis are documented annually with over 60,000 deaths worldwide [6]. While the incidence of leptospirosis is on the rise in companion animals, an accurate count of cases per annum is elusive [7]. In dogs, cases are most common in unvaccinated dogs <6 months of age and <14 lbs in weight with approximately 25% of cases being fatal (American Kennel Club; https://www.akcchf.org/educational-resources/library/an-update-on-canine.html; accessed on 01/07/2021).

In veterinary medicine (small and large animal) vaccination serves as the primary preventative strategy for leptospirosis [8]. *Leptospira* bacterin vaccines consist of inactivated cell lysates of multiple strains. The antibody response to *Leptospira* bacterin vaccines is directed largely at LPS. As a result, the protection afforded by bacterins is limited primarily to the serovars represented in the vaccine formulations [9], [10]. The dominant circulating serovars in the United States are Icterohaemorrhagiae, Canicola, Grippotyphosa, Pomona, Bratislava, and Autumnalis. In Europe, Icterohaemorrhagiae, Grippotyphosa, Australis, Sejroe and Canicola are dominant [11]. In addition to limited cross-protection [12], concerns have also been raised about the reactogenicity of *Leptospira* bacterin vaccines, particularly those used in canines [13], [14]. Specific protein antigens that convey broad protection remain to be identified (reviewed in [9]). The development of recombinant protein based subunit vaccines represents an attractive alternative to bacterin based vaccines.

In this study we assessed the vaccine potential of the *L. interrogans* serovar Copenhageni strain Fiocruz L1-130 general secretory protein D (GspD) [15]. GspD is a 67 kDa protein that is predicted to function as a secretin for a leptospiral type 2 secretion (T2S) system [16], [17]. Here we demonstrate that GspD is an outer membrane antigen produced during *in vitro* cultivation and during natural infection in canines. Immunization of rats with recombinant GspD (GspD) elicited potent bactericidal antibody that effectively targeted multiple *Leptospira* species, strains and serovars of the P1 subclade. Antibody mediated killing was demonstrated to occur through a complement independent mechanism. Epitope mapping revealed that the bactericidal epitopes of GspD reside within its N-terminal ‘N0′ domain [18], [19]. The data presented within support the potential use of GspD in a subunit-based vaccine formulation.

## 2 Materials and methods

### 2.1 Bacterial strains and cultivation

*Leptospira* isolates were cultured at 30 °C in Ellinghausen-McCullough-Johnson-Harris (EMJH) medium supplemented with Probumin vaccine grade BSA (Millipore) as previously described [20]. Growth was monitored by wet-mounts using dark-field microscopy. All isolates assessed *in vitro* or *in silico* are described in Table 1. Note that strain virulence was not assessed as part of this study.

**Table 1:** Bacterial isolates included in this study.

Isolate	Source	GspD Amino Acid Access No. a
*Leptospira interrogans* sv. Copenhageni str. Fiocruz L1-130	Human, Brazil	AAS70166.1
*Leptospira interrogans* sv. Lai str. 56601	Human, China	AAN49574.2
*Leptospira interrogans* sv. Canicola str. LT1962	Human, Taiwan	EMF70412.1
*Leptospira interrogans* sv. Canicola str. Mex1	*Unknown*	Not determined
*Leptospira interrogans* sv. Canicola str. Kito	Canine, Brazil	EMK18369.1
*Leptospira interrogans* sv. Pomona str. Kennewicki LC82-25	Human, USA	EJO79568.1
*Leptospira interrogans* sv. Manilae str. L495	Human, Philippines	SOR62965.1
*Leptospira noguchii* sv. Autumnalis str. Bonito	Human, Brazil	EMI64742.1
*Leptospira noguchii* sv. Autumnalis str. ZUN142	Human, Peru	EMO42106.1
*Leptospira kirschneri* sv. Grippotyphosa str. RM52	Swine, USA	EJO70340.1
*Leptospira alstonii* sv. Sichuan str. 79601	Frog, China	EMJ95582.1
*Leptospira alstonii* str. New Haven	Freshwater, USA	Not determined
*Leptospira santarosai* sv. Shermani str. LT 821	Human, Taiwan	WP_004460673.1
*Leptospira borgpetersenii* sv. Hardjo-bovis str. Sponselee	Cattle, Netherlands	WP_002751252.1
*Leptospira borgpetersenii* sv. Ballum str. M2	Mouse, Puerto Rico	Not determined
*Leptospira weilii* sv. Topaz str. LT2116	Human, Thailand	WP_004503249.1
*Leptospira wolffii* sv. Khorat str. Khorat-H2	Human, Thailand	EPG66587.1
*Leptospira licerasiae* sv. Varillal str. VAR 010	Human, Peru	EIE01661.1
*Leptospira meyeri* sv. Hardjo str. Went 5	*Unknown*, Canada	EKJ85129.1
*Leptospira yanagawae* sv. Saopaulo str. Sao Paulo	Freshwater, Brazil	EOQ90642.1
*Leptospira biflexa* sv. Patoc strain Patoc 1 (Paris)	Freshwater, Italy	ABF47508.1
*Leptonema illini* DSM 21528^b^	Cattle, USA	EHQ05871.1
*Turneriella parva* DSM 21527^b^	N/A	AFM14754.1
*Escherichia coli* str. K-12 substr. MG1655 b	N/A	NP_417784.4

a All Isolates were provided by Drs. James Matsunaga, David Hatke (UCLA), Elsio Wunder and Albert Ko (Yale University).

b GspD amino acid sequences of *L. illini*, *T. parva* and *E*. *coli* str. K-12 substr. MG1655 served as outliers in the phylogenetic analyses

### 2.2 Phylogenetic analyses

GspD amino acid sequences from diverse *Leptospira* isolates were aligned using Mega X [21]. GspD homologs from *Leptonema illini, Turneriella parva,* and *Escherichia coli* K-12 served as outliers. A phylogenetic tree was constructed in Mega X using the neighbor-joining tree algorithm with 500 bootstraps, a poisson model of amino acid substitution, and pairwise deletion of gaps [21]. Mega X was also used to calculate percent amino acid identity and similarity values. GspD accession numbers are listed in Table 1.

### 2.3 Cloning, expression and purification of recombinant proteins

PCR and cloning were performed using standard conditions as previously described [22]. *LipL32, gspD, qlp42* and *gspD* gene fragments were PCR amplified from *L. interrogans* serovar Copenhageni strain Fiocruz L1-130 genomic DNA using *Phusion* polymerase (Thermo Scientific) and primers that harbor restriction sites for cloning (Table 2). Signal sequences predicted by SignalP-5.0 [23] were excluded from the amplified *lipL32* and *qlp42* sequences. The amplicons were purified using QIAquick PCR Purification kits (Qiagen), digested with the appropriate restriction enzymes (New England Biolabs), ligated with linearized pET-45b(+), transformed into *E. coli* BL21(DE3) cells, and protein production induced with 1.0 mM Isopropyl β-d-1-thiogalactopyranoside [24]. All recombinant proteins were produced with an N-terminal hexa-histidine tag. Gene sequences were verified on a fee for service basis (Genewiz). Recombinant proteins that separated with the soluble fraction (Qlp42 and LipL32) were purified by nickel affinity chromatography using an ÄKTA purification platform as previously described [25]. Recombinant *Treponema denticola* Factor H binding protein B (FhbB), generated as part of an earlier study, served as control in several experiments [26]. Recombinant proteins that partitioned in the insoluble fraction (GspD and GspD sub-fragments F1 through F5) were purified using urea-denaturing conditions and then dialyzed stepwise into 1X PBS [24]. Protein concentrations were determined by bicinchoninic acid assay (BCA; Pierce).

**Table 2:** Oligonucleotide primer sequences.^a^

Primer name	Primer sequence (5′–3′)	Amplicon b
*lipL32-Fwd*	ATAGTCACCGGTGCTTTCGGTGGTCTGCCAAG	22–272
*lipL32-Rev*	ATAGTCCGGCCGTTACTTAGTCGCGTCAGAAGCAG	22–272
*qlp42-Fwd*	ATAGTCGGATCCCGCTTGTAAGAAACCTACCGAAAGTTCC	21–388
*qlp42-Rev*	ATAGTCCGGCCGTTATCAGAATTTAGCTTTTGTTTGGAAGTC	21–388
*gspD-FL-Fwd*	ATAGTCGGATCCCATGTCCGGAACAATCAGTCAACTTTC	1–596 (Full-length)
*gspD-FL-Rev*	ATAGTCCGGCCGTTAATCCCCTCTTTCTCTGATTTCTCTTTC	1–596 (Full-length)
*gspD-F1-Fwd*	ATAGTCGGATCCCTGGTGGATGTCCGGAACAATC	1–135 (Fragment F1)
*gspD-F1-Rev*	ATAGTCCGGCCGTTAAGATCTAGCAAGTGCATCTTTAATTTTAACAATCG	1–135 (Fragment F1)
*gspD-F2-Fwd*	ATAGTCGGATCCCTGGTGGGTAGTAGAAGAACCGG	115–250 (Fragment F2)
*gspD-F2-Rev*	ATAGTCCGGCCGTTATAACTTAACTAAAGTCGCCGCGATTTTTTC	115–250 (Fragment F2)
*gspD-F3-Fwd*	ATAGTCGGATCCCTGGTGGCATATTTATACTTTGGAATACAGTG	230–365 (Fragment F3)
*gspD-F3-Rev*	ATAGTCCGGCCGTTAATTGACCTGACCGTTCGAATTGATAATATTC	230–365 (Fragment F3)
*gspD-F4-Fwd*	ATAGTCGGATCCCTGGTGGCAATTCAATTCTGGTCTTTC	345–480 (Fragment F4)
*gspD-F4-Rev*	ATAGTCCGGCCGTTAAATCTCTGCGATATTTTTAATCTCCTGAAAGAGTTC	345–480 (Fragment F4)
*gspD-F5-Fwd*	ATAGTCGGATCCCTGGTGGAACAAAAATAATAAGATCACTCTCGAAC	460–596 (Fragment F5)
*gspD-F5-Rev*	ATAGTCCGGCCGTTAATCCCCTCTTTCTCTGATTTCTCTTTCTTTG	460–596 (Fragment F5)

a Sequences included for ligation into pET-45b(+) are underlined.

b Amino acid regions encoded by amplicon based on *L. interrogans* sv. Copenhageni str. Fiocruz L1-130 sequence (GenBank Accession No. NC_005823.1).

### 2.4 Generation of antisera

Antisera against recombinant GspD, Qlp42 and LipL32 were generated as previously described [27]. In brief, Sprague Dawley rats were immunized (intraperitoneally) with 50 µg of recombinant protein in Freund’s complete adjuvant (Sigma-Aldrich) with 2 boosts of 25 µg recombinant protein in Freund’s incomplete adjuvant (Sigma-Aldrich) delivered 2 weeks apart. One week after the final boost, rats were sacrificed, blood collected via cardiac puncture and the sera harvested using Grenier Bio-one Vaccuette Z Serum Sep Clot Activator tubes (Grenier). All animal use protocols were reviewed and approved by the VCU IACUC and followed the Guide for the Care and Use of Laboratory Animals (Eighth edition).

### 2.5 SDS-PAGE, immunoblot, and dot-blot analyses

Cell lysates (0.3 OD_600_ units per lane) were separated using AnykD Criterion Precast Gels (Bio-Rad) and transferred to PVDF membranes using the Transblot Turbo System per the manufacturer’s protocol (Bio-Rad). Dot-blots were generated by spot application of 500 ng of GspD, LipL32, and FhbB (negative control) on to nitrocellulose membranes (22 µM pore size; BioRad) followed by air drying. Immunoblots and dot-blots were incubated with blocking buffer (PBS with 5% nonfat dried milk; 0.2% Tween 20; 2 h). Microscopic agglutination test (MAT) positive canine sera (1:200 dilution) were added (1 h), the blots were washed and rabbit anti-canine IgG HRP-conjugated secondary antibody (1:20000 dilution; Novus Biologicals) was added. IgG binding was detected using ECL substrate (Bio-Rad) and images were captured using a ChemiDoc Touch Imaging System (Bio-Rad). Note that the immunoblot and dot-blot images were cropped to generate the figures.

### 2.6 Triton X-114 extraction and phase partitioning

Triton X-114 extraction and phase partitioning were performed as previously described [28] with some modifications. Approximately 10^11^ mid-log phase *L. interrogans* serovar Copenhageni strain Fiocruz L1-130 cells were recovered by centrifugation, washed twice (1X PBS, 5 mM MgCl_2,_ 1% protease inhibitor cocktail (PIC; Sigma-Aldrich). Lysate was diluted to OD_600_ = 3.0 and pelleted by centrifugation (3000*g*, 15 min, 4 °C). The pellet was resuspended in 1 mL extraction buffer (150 mM NaCl, 1 mM EDTA, 10 mM Tris-HCl, 1% Triton X-114, 1% PIC), incubated on ice (2 hr) and centrifuged (17,000*g*, 10 min, 4 °C) to separate the detergent soluble and insoluble fractions. The supernatant was pipetted into a separate tube and 20 mM CaCl_2_ was added, followed by incubation (37 °C, 15 min) and centrifugation (2000*g*, 10 min). The aqueous supernatant was decanted into a separate tube and the pelleted detergent phase was washed twice with PBS and resuspended. Aqueous and detergent phases were acetone precipitated with 10X volumes of ice-cold acetone. Precipitate was collected by centrifugation (12,600*g*, 30 min, 4 °C) and resuspended in PBS.

### 2.7 Bactericidal assays

Bactericidal activity of anti-GspD antisera was assessed as previously described [27], [29] with some modifications. In brief, 4 µL of mid-log phase *Leptospira* cultures (density of ~100 cells per field of view under 400× magnification) were combined with 8 µL of EMJH media. Four µL of heat-inactivated (HI; 56 °C, 30 min) rat anti-GspD antiserum, 4 µL of complement certified normal human serum (NHS; Innovative) or 4 µL of HI-NHS was added. All assays were performed in triplicate. Cells incubated in EMJH, NHS, and rat pre-immune (PI) serum served as negative controls. The samples were incubated at 30 °C for 2 h, and the average number of live cells per 5 fields of view determined by visual counting under dark-field microscopy. Percent killing was calculated by determining the decrease in the number of live cells in the treatment groups versus the NHS + rat pre-immune serum negative control. Statistical analyses were conducted using Student’s *t* test.

### 2.8 Epitope localization

Recombinant proteins (500 ng) were coated onto 96-well plates (Corning) in triplicate and screened with serum (1:100) from nine client-owned, MAT positive canines [30]. Secondary antibody (rabbit anti-canine IgG HRP-conjugated; Novus Biological; 1:15000) was added and absorbance measured at 405 nm using a microtiter plate reader (BioTek). To localize the bactericidal epitopes, anti-GspD antiserum was adsorbed (overnight; 30 °C) with 250 ng µL^−1^ of full-length (FL) GspD, GspD fragment (F) 1, GspD F2, GspD F3, GspD F4, GspD F5 (Table 2), or FhbB [26]. Bactericidal activity was assessed as detailed above. Cells incubated with rat PI serum alone and non-adsorbed anti-GspD antiserum alone served as negative and positive controls, respectively. The *Treponema denticola* FhbB protein served as an additional negative control [31].

## 3 Results and discussion

### 3.1 Analysis of GspD conservation amongst Leptospira species and strains

**Fig. 1:**
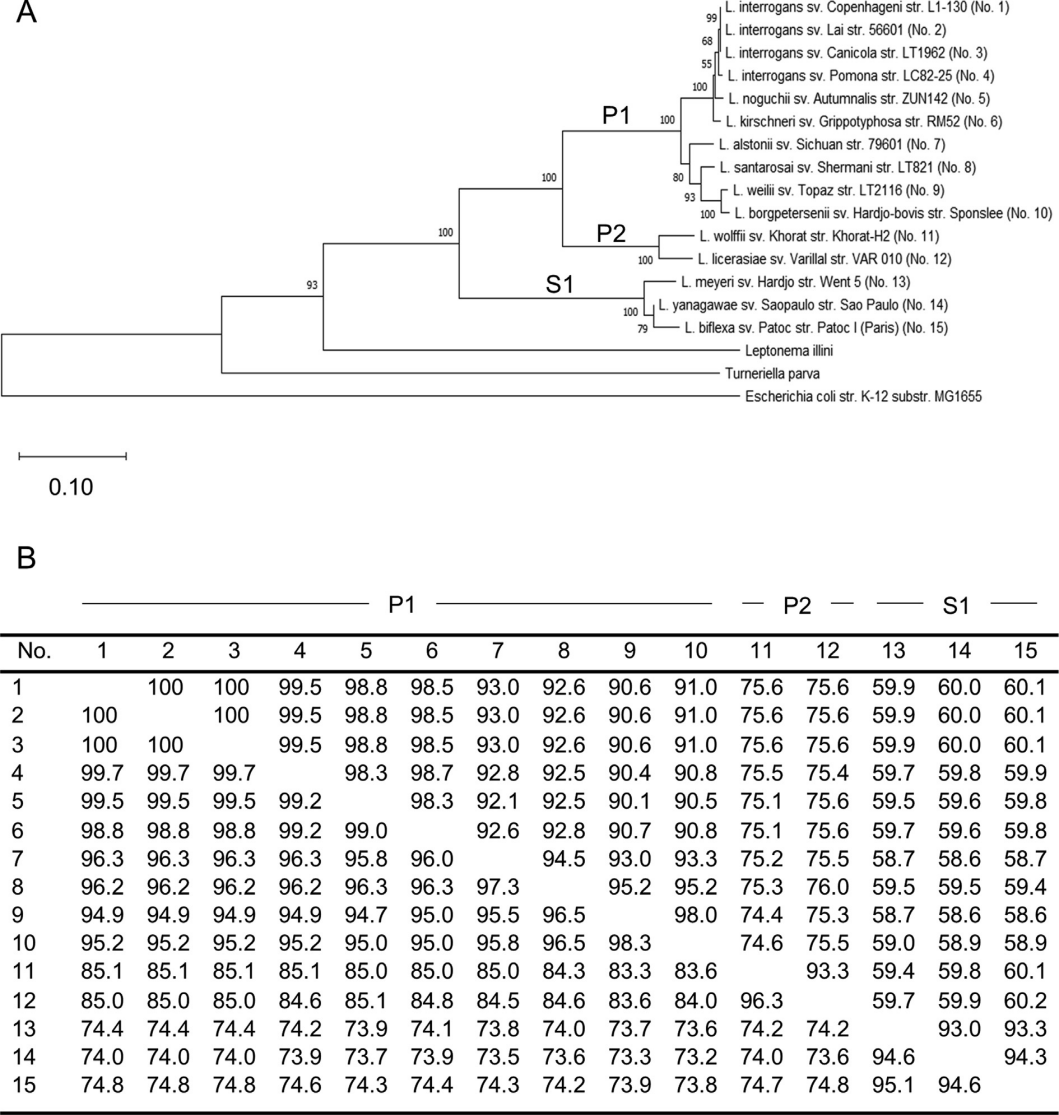
Phylogenetic analyses and pairwise sequence comparisons of GspD amino acid sequences. Panel A presents a phylogenetic tree constructed using GspD amino acid sequences as detailed in the text. Representative GspD sequences from *Leptospira* subclades P1, P2, and S1 (as indicated) were included. *Leptonema illini, Turneriella parva* and *Escherichia coli* str. K-12 served as outgroups. The results of bootstrap analyses are indicated on each node and the scale bar is shown in the lower left. Note that the numbers (No.) in parentheses that follow each isolate designation correspond to those used in the distance matrix table (Panel B). Panel B presents the GspD percent amino acid similarity and identity values (lower and upper quadrants, respectively). The subclade classification of each isolate of origin is indicated along the top of panel B. All accession numbers can be found in Table 1.

Evolutionary distances and phylogenetic relationships among GspD sequences were determined through pairwise comparisons. A phylogenetic tree is presented in Fig. 1A. The *Leptospira* GspD sequences assessed divide into three distinct phyletic clusters that are well-supported by bootstrap analyses. Amino acid identity and similarity values are presented in Fig. 1B. Identity values among *Leptospira* P1 subclade isolates ranged from 90.1 to 100%. GspD sequences derived from isolates of subclades P2 and S1 display approximately 75 and 60% identity with P1 subclade isolates, respectively. Hence, while GspD is conserved within a subclade, divergence between subclades is significant.

### 3.2 *In vitro* expression and subcellular localization of GspD

**Fig. 2:**
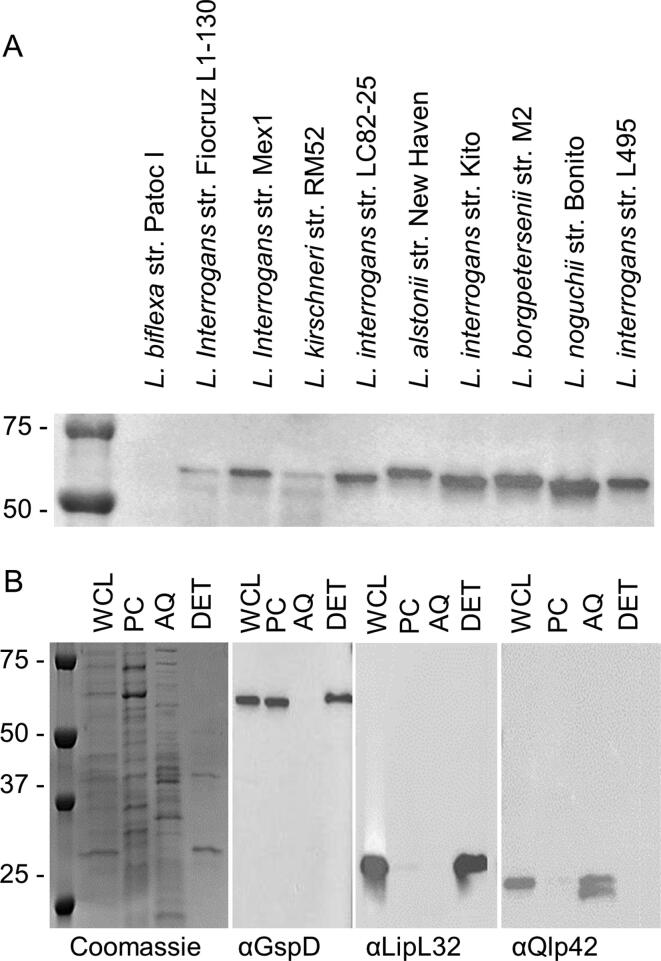
In vitro expression and cellular localization of GspD. Panel A presents an immunoblot in which whole cell lysates of diverse *Leptospira* isolates were screened with anti-GspD antiserum as detailed in the text. In panel B, *L. interrogans* serovar Copenhageni strain Fiocruz L1-130 was subjected to Triton X-114 extraction and phase partitioning. The resulting fractions were separated by SDS-PAGE, transferred to PVDF membranes and screened with antiserum (1:1000 dilution) as indicated below each immunoblot. The left panel of Panel B presents a Coomassie Brilliant Blue stained SDS-PAGE gel. Antisera against LipL32 and Qlp42 served as partitioning controls for outer membrane and periplasmic proteins, respectively. Abbreviations are as follows: whole cell lysate (WCL), protoplasmic cylinder (PC), aqueous phase (AQ), and detergent phase (DET). Molecular weight standards are indicated to the left. All methods were as detailed in the text.

*In vitro* expression of GspD by diverse P1 strains was demonstrated by screening immunoblots of *Leptospira* whole cell lysates with anti-GspD antiserum. A single protein of approximately 67 kDa was detected in all strains tested with the exception of the saprophyte, *L. biflexa strain Patoc I,* which belongs to subclade S1 (Fig. 2A). While it is possible that *L. biflexa* does not produce GspD *in vitro*, an alternative possibility is that its GspD protein, which shares only 60% amino acid identity with the *L. interrogans* serovar Copenhageni strain Fiocruz L1-130 GspD protein used to generate the sera, is antigenically distinct. In light of the conservation of GspD at the intra-clade level, the difference in signal intensity among P1 subclade strains observed in the immunoblot most likely suggests variable levels of expression *in vitro* (Fig. 2A).

The subcellular localization of GspD was determined through Triton X-114 extraction and phase partitioning of *L. interrogans* serovar Copenhageni strain Fiocruz L1-130. The resulting fractions were immunoblotted and screened with anti-GspD, anti-LipL32 and anti-Qlp42 antisera (Fig. 2B). GspD was detected in the whole cell lysate, the detergent insoluble phase (protoplasmic cylinder), and the detergent soluble phase (outer membrane). Based on earlier studies that demonstrated that GspD homologs of other bacterial species interact with both the inner and outer membrane [18], [19], [32], the detection of GspD in both the detergent insoluble and soluble phases was expected and is consistent with its putative function as a secretin in type 2 secretion [17]. As controls, identical immunoblots were screened with anti-LipL32 and anti-Qlp42 antisera. LipL32 and the Qlp42 have been demonstrated to partition with the outer membrane and aqueous phases, respectively [28], [33], [34], [35], [36]. Both proteins partitioned as expected.

### 3.3 GspD is expressed during natural infection in client-owned canines

**Fig. 3:**
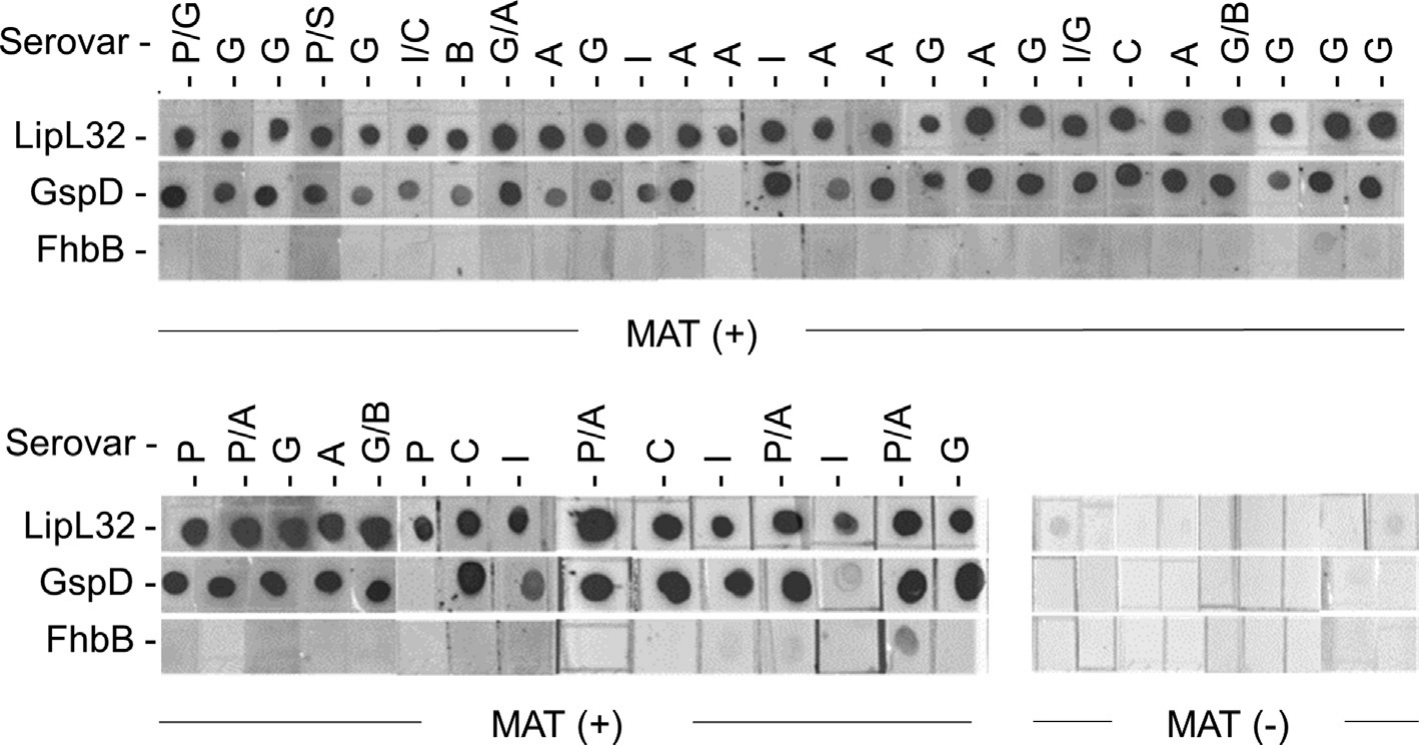
GspD is antigenic during natural infection in client-owned canines. Dot-blots of full length recombinant proteins (indicated to the left) were screened with sera from MAT positive and MAT negative client owned dogs (1:200 dilution) as indicated below each panel. For each serum sample, the serovar(s) that yielded the the highest MAT titers are indicated above each dot-blot. Abbreviations were as follows: Pomona (P), Icterohaemorrhagiae (I), Canicola (C), Grippotyphosa (G), Hardjo-bovis (H), Bratislava (B), Autumnalis (A), and Sejroe (S). For sera that had equal titers to two serovars, both serovar abbreviations are provided. LipL32 was included as a positive control and the *T. denticola* FhbB protein served as a negative control. Molecular weight standards are indicated on the left.

Serum from forty-one MAT positive and nine MAT negative client owned dogs were screened for IgG to GspD using a dot-blot format. Recombinant LipL32 and FhbB (*T. denticola* FhbB [26]) served as positive and negative control detection antigens, respectively. Of the forty-one MAT positive dogs, 92.7% were IgG positive for antibody to GspD and all were immunoreactive with LipL32, a known surface exposed *in vivo* antigen (Fig. 3) [28], [33]. MAT negative sera were IgG negative for antibody to all proteins tested and all sera were antibody negative for FhbB. The detection of antibodies to GspD in canines is consistent with the detection of anti-GspD antibodies in the sera of confirmed human leptospirosis patients [37]. It can be concluded that GspD is actively produced during *Leptospira* infections in canines.

### 3.4 Anti-GspD antiserum is bactericidal and kills through a complement independent mechanism

**Fig. 4:**
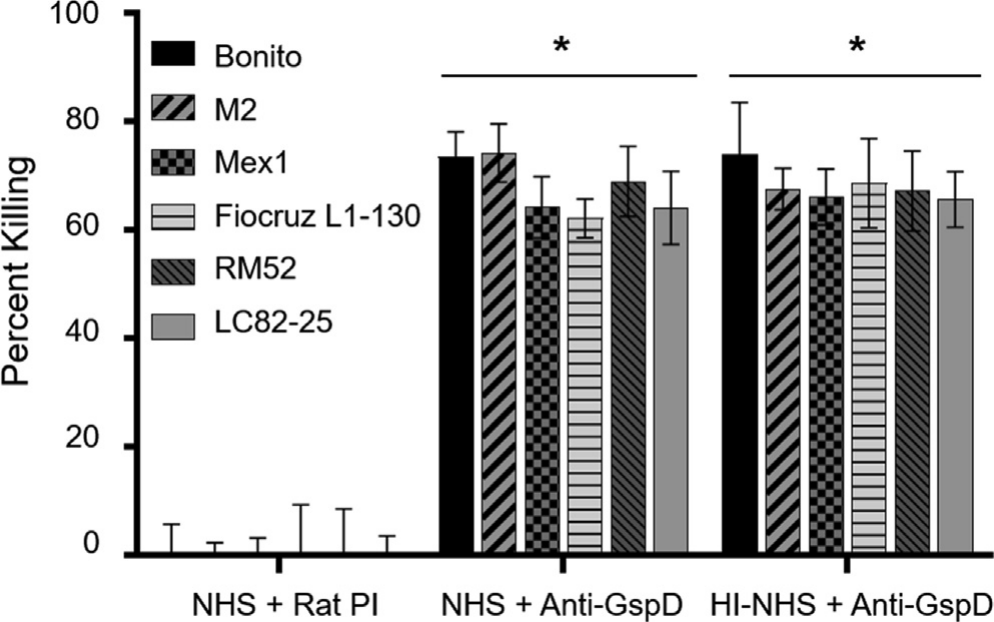
Anti-GspD antisera is bactericidal and kills diverse strains through a complement independent mechanism. The bactericidal activity of rat anti-GspD antiserum against diverse *Leptospira* strains (as indicated in the figure key) was assessed as described in the methods. Abbreviations are as follows: NHS (complement certified normal human serum), HI-NHS (heat inactivated normal human sera), and Rat PI (rat pre-immune serum). Equivalent numbers of cells were incubated with NHS, HI-NHS, rat PI, or anti-GspD antisera as indicated below the figure. Percent killing was determined by visual examination of cell numbers per field of view using dark field microscopy as detailed in the text. Differences in cell counts per field of view in the negative control (NHS + rat PI) versus in the treatment groups (NHS + Anti-GspD and HI-NHS + Anti-GspD) were assessed using the two-tailed student’s *t-*test (* indicates *P* ≤ .05).

Bactericidal assays revealed that anti-GspD antibody can kill diverse species and strains of *Leptospira* of the P1 subclade (Fig. 4). Antibody dependent killing (60–74%) occurred independent of complement (Fig. 4) with no significant difference in killing in the presence of active NHS vs HI-NHS (*P* = .41). The mechanism of killing by anti-GspD antibody remains to be determined but could be linked to the critical functions mediated by secretin proteins and T2SS in general. The binding of antibody to GspD may interfere with the passage of effector molecules from the cell into the extracellular milieu [38]. Importantly, anti-GspD antibody effectively killed all strains tested including those of differing serotypes suggesting that GspD or domains derived from it, may be good candidates for inclusion in a multi-protein subunit based vaccine.

### 3.5 Localization of the bactericidal epitopes of GspD

**Fig. 5:**
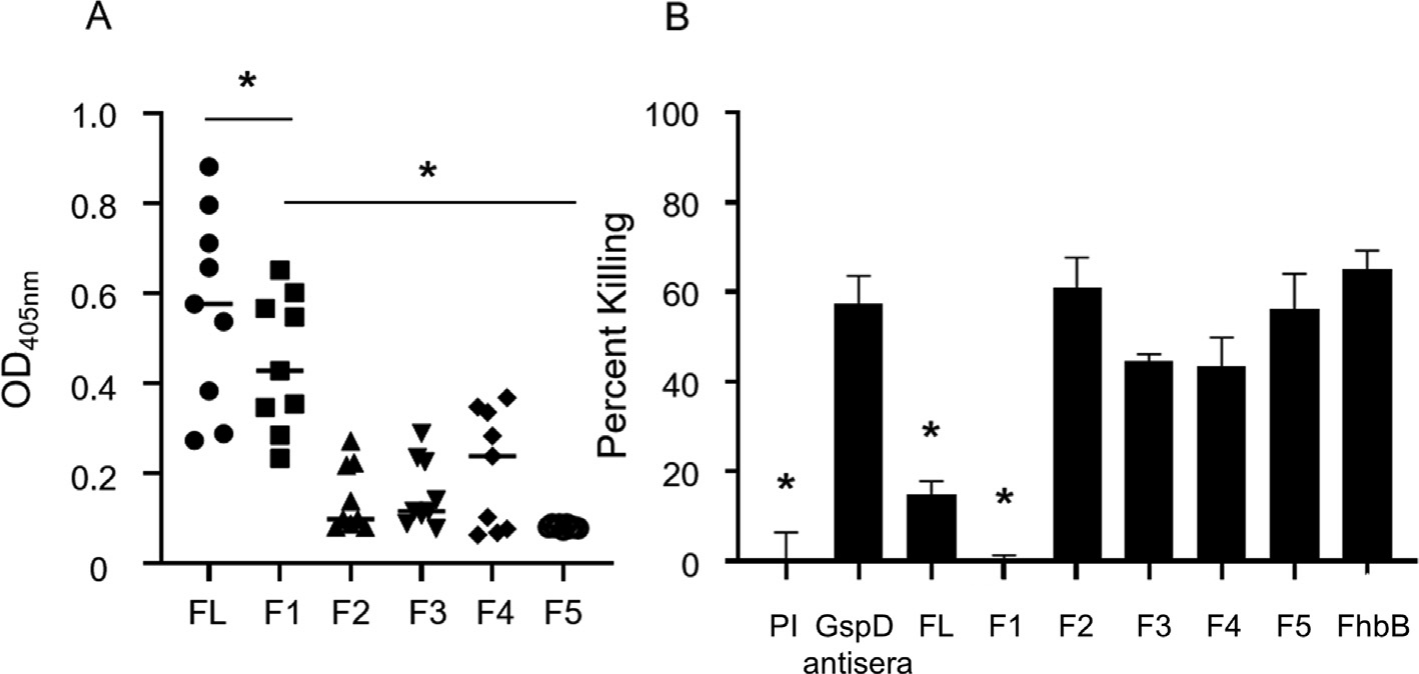
Localization of the bactericidal epitopes of GspD to the N-terminal domain. In panel A, full-length (FL) GspD and sub-fragments (indicated as F1 through F5) were screened with serum from nine individual MAT + canines (1:100 dilution) by ELISA. IgG levels to each protein were determined as detailed in the text. The data were assessed using the two-tailed student’s *t* test based on the mean value for each group (* indicates *P* < .05). In panel B, anti-GspD antisera was adsorbed with full length recombinant GspD FL or the F1 through F5 fragments (as indicated along the x axis). The adsorbed sera were then used in bactericidal assays in the presence of complement preserved normal human serum (NHS) and percent killing was determined as described in the text. Cells incubated with NHS and rat pre-immune (PI) serum alone served as a negative control. Cells incubated with NHS and non-adsorbed anti-GspD antiserum (GspD antisera) served as a positive control. Significance was evaluated by comparing cells per field of view of each treatment group with the GspD antisera alone control sample using the two-tailed student’s *t* test (* indicates *P* < .05).

To localize the immunodominant epitopes of GspD that are presented during natural infection, GspD fragments harboring 20 amino acid overlaps were generated (Table 2) and screened with MAT positive canine sera by ELISA (Fig. 5A). The sera reacted most strongly with full length GspD and the F1 fragment (residues 1–135). IgG binding to other fragments was low and variable among individual dogs. The difference in the level of antibody binding to F1 versus all other fragments was significant (*P* ≤ 0.0001). The results demonstrate that the immunodominant epitopes of GspD reside within a segment of the N-terminal N0 domain within amino acids 1 through 135.

To verify that the epitopes of GspD that elicit bactericidal antibody reside within the F1 fragment, bactericidal assays were performed with anti-GspD antisera absorbed with full length GspD, GspD sub-fragments or FhbB prior to conducting the assay (Fig. 5B). Bactericidal activity was significantly reduced by both full length GspD or the F1 fragment (P < 0.01), but not by adsorption with fragments F2, F3, F4, F5 or the irrelevant FhbB antigen. Hence, it can be concluded the bactericidal epitopes of GspD reside within the first 135 amino acids which comprise a majority of the N0 domain. The N0 domain extends from the outer membrane across the periplasm where it interacts with substrates and inner membrane components [19]. Antibody binding to the N0 domain may disrupt T2S and thus explain the basis for the complement independent bactericidal activity of anti-GspD antibody. A BLASTP search using GspD amino acid residues 1 through 135 as the query revealed that the N0 domain of GspD is highly conserved among isolates of subclade P1. This is noteworthy as subclade P1 isolates exhibit greater genome diversity at the intra-clade level than isolates belonging to subclades P2, S1, and S2, and are most commonly associated with severe clinical manifestations of leptospirosis [3].

## 4 Conclusions

In this study we have demonstrated that the *Leptospira* secretin protein, GspD, is expressed *in vitro* and antigenic during natural infection in canines. Recombinant GspD delivered to rats elicited a high titer IgG antibody response. *In vitro* assays demonstrated that anti-GspD antibodies are bactericidal and kill through a complement-independent mechanism. The epitopes that elicit bactericidal antibodies were localized within the within the N0 domain of GspD that spans the N-terminal 135 residues. Anti-GspD antibody displayed significant killing activity against antigenically diverse subclade P1 strains including *L. interrogans* serovars Canicola, Copenhageni and Pomona*, L. kirschneri* serovar Grippotyphosa*, L. noguchii* serogroup Autumnalis*,* and *L. borgpetersenii* serogroup Ballum. While GspD is conserved amongst subclade P1 isolates, the GspD sequences of other subclades show significant divergence. Hence, a GspD vaccine formulation may need to be multivalent, including the F1 fragment of at least one P1 and P2 subclade isolate. While this study was in review, it was demonstrated that *L. interrogans* serovar Canicola (LOCaS46) (subclade P1) full length GspD conveyed partial protection to hamsters against lethal challenge with the homologous strain of *Leptospira* [39]. The data presented here further support the potential utility of GspD as a component of a subunit vaccine for leptospirosis.

A few studies have sought to develop recombinant chimeric antigens that can elicit antibody against multiple *Leptospira* antigens [40], [41], [42], [43], [44]. An OmpL1-LipL32-LipL21 chimeric conferred significant protection against lethal homologous challenge with *L. interrogans* sv. Lai in hamsters [45] and a LigAc-LenA-LcpA-Lsa23 significantly reduced leptospiral renal burden [46]. However, the potential for these chimeric protein vaccinogens to elicit protection against heterologous strains has not yet been assessed. Recently, a recombinant chimeric epitope based vaccine antigen, referred to as a chimeritope, was successfully developed as a vaccine antigen for canine Lyme disease. The sequences encoding the immunodominant and variable L5 and H5 epitopes from several *Borreliella burgdorferi* outer surface protein C variants were linked to form a single contiguous gene sequence. Protein expressed from this chimeritope encoding sequence, designated as Ch14, was demonstrated to elicit broadly cross reactive and protective antibodies [27], [47]. The Ch14 chimeritope is one of two recombinant antigens that comprise the commercially successful canine Lyme disease vaccine, VANGUARD®crLyme (Zoetis) [47], [48]. The remarkable diversity of the *Leptospira* suggests that a chimeritope approach may offer a path to a broadly protective vaccine antigen for both human and veterinary leptospirosis. Future work will seek to incorporate epitopes several leptospiral antigens into a single recombinant chimeritope protein.

## Declaration of Competing Interest

The authors declare that they have no known competing financial interests or personal relationships that could have appeared to influence the work reported in this paper.
